# Moderate intensity aerobic exercise may enhance neuroplasticity of the contralesional hemisphere after stroke: a randomised controlled study

**DOI:** 10.1038/s41598-023-40902-2

**Published:** 2023-09-02

**Authors:** Gabrielle Hill, Finn Johnson, Jeric Uy, Ines Serrada, Beben Benyamin, Maayken Van Den Berg, Brenton Hordacre

**Affiliations:** 1 Clinical Rehabilitation, College of Nursing and Health Sciences, Flinders University, Adelaide, 5042 Australia; 2 Allied Health and Human Performance, University of South Australia, Adelaide, 5000 Australia; 3 Australian Centre for Precision Health, Allied Health and Human Performance, University of South Australia, Adelaide, 5000 Australia; 4 South Australian Health and Medical Research Institute, Adelaide, 5000 Australia; 5 Innovation, IMPlementation and Clinical Translation (IIMPACT) in Health, University of South Australia, City East Campus, GPO Box 2471, Adelaide, 5001 Australia

**Keywords:** Medical research, Neurological disorders

## Abstract

Upregulation of neuroplasticity might help maximize stroke recovery. One intervention that appears worthy of investigation is aerobic exercise. This study aimed to determine whether a single bout of moderate intensity aerobic exercise can enhance neuroplasticity in people with stroke. Participants were randomly assigned (1:1) to a 20-min moderate intensity exercise intervention or remained sedentary (control). Transcranial magnetic stimulation measured corticospinal excitability of the contralesional hemisphere by recording motor evoked potentials (MEPs). Intermittent Theta Burst Stimulation (iTBS) was used to repetitively activate synapses in the contralesional primary motor cortex, initiating the early stages of neuroplasticity and increasing excitability. It was surmised that if exercise increased neuroplasticity, there would be a greater facilitation of MEPs following iTBS. Thirty-three people with stroke participated in this study (aged 63.87 ± 10.30 years, 20 male, 6.13 ± 4.33 years since stroke). There was an interaction between Time*Group on MEP amplitudes (*P* = 0.009). Participants allocated to aerobic exercise had a stronger increase in MEP amplitude following iTBS. A non-significant trend indicated time since stroke might moderate this interaction (*P* = 0.055). Exploratory analysis suggested participants who were 2–7.5 years post stroke had a strong MEP facilitation following iTBS (*P* < 0.001). There was no effect of age, sex, resting motor threshold, self-reported physical activity levels, lesion volume or weighted lesion load (all *P* > 0.208). Moderate intensity cycling may enhance neuroplasticity in people with stroke. This therapy adjuvant could provide opportunities to maximize stroke recovery.

## Introduction

Stroke remains a leading global cause of adult disability^1^, with extensive rehabilitation often required to support recovery. While recovery remains possible years after a stroke, behavioral evidence suggests the rate of improvement is more rapid within the first few months^2^. These early gains are thought to be underpinned by a spontaneous, time-limited, period of heightened neuroplasticity^3, 4^. In support, preclinical data indicates that delays to the initiation of therapy, missing the critical period of heightened neuroplasticity, results in poorer recovery^5^. It appears therapy that coincides with periods of heightened neuroplasticity is more likely to promote maximal recovery.

A topical question in stroke recovery is whether it is possible to re-open, or prolong, the spontaneous period of enhanced neuroplasticity seen after stroke. Ability to do so might lead to greater recovery. A mouse model of stroke has provided some evidence to suggest this may be possible^6^. Following an initial ischemic event, where therapy was delayed and recovery incomplete, mice were exposed to a second ischemic event to re-establish a period of enhanced plasticity. Subsequent training led to full recovery from the previous stroke. While not feasible in humans, early pharmacology studies investigating neuroplasticity promoting drugs reported enhanced recovery in human chronic stroke survivors^7^. However, more recent results are less conclusive^7^. Alternatively, there is some evidence that cardiovascular exercise might facilitate neuroplasticity. Moderate intensity exercise has demonstrated promising results for increasing neuroplasticity in rodent stroke models^8^, but low intensity exercise did not induce neural changes or promote neuroplasticity in humans^9^. More intensive aerobic exercise, such as high intensity interval training, has beneficial effects on modulating neuroplasticity in both healthy adults^10^ and people with stroke^11–13^. However, high intensity exercise for people with stroke can be challenging^14^. Stroke survivors often present with multiple co-morbidities that may pose a greater risk for participation in high intensity exercise. This could limit the feasibility of future therapeutic clinical trials or clinical implementation. Furthermore, if the therapeutic rationale is to increase neuroplasticity with aerobic exercise and subsequently perform training to facilitate recovery, it is reasonable to consider some patients may experience fatigue from high intensity exercise, limiting capacity for therapy. Moderate levels of exercise intensity might be considered safer^15^, better tolerated and could still offer benefits of upregulated neuroplasticity as observed in preclinical studies^8^. In humans, there is evidence that 20–30 min of moderate intensity exercise increased brain derived neurotrophic factor (BDNF)^16^, promoted region specific increases in cerebral blood flow^17^, and improved behavioral performance^18^. The effects of moderate intensity exercise on brain neuroplasticity appear worthy of investigation.

The aim of this pilot study was to investigate whether moderate intensity exercise could increase neuroplasticity in people with stroke. We specifically investigated people who were several months after stroke to avoid the initial, brief, spontaneous period of enhanced neuroplasticity that emerges early after stroke^3^. To evaluate capacity for neuroplasticity, we used a repetitive stimulation protocol, known as intermittent theta-burst stimulation (iTBS) which has been shown to modulate the efficiency of synapses within the cortex^19^. Physiologically, this can be quantified as a change in cortical excitability. Therefore, the hypothesis was that if moderate intensity exercise increases capacity for neuroplasticity, then the physiological response to iTBS would be greater compared to people who do not undertake exercise. Given the challenges of performing brain stimulation on the stroke affected hemisphere, and that exercise is likely to have a global effect on brain physiology, we assessed neuroplasticity from the contralesional hemisphere. If moderate intensity exercise does increase neuroplasticity in people with stroke, then it might provide one method to explore as a technique to re-open a period of enhanced neuroplasticity. Future trials could use exercise as a brain priming therapy to increase responsiveness to rehabilitation.

## Methods

### Participants

People who had experienced stroke at least three months prior, were community ambulators, medically stable and over the age of 18 years were invited to participate. Recruitment occurred via advertisement in a university health clinic and distributing information to willing volunteers in a research database. Exclusion criteria were previous diagnosis of another neurological disease, recent craniotomy or neurosurgical intervention, any concurrent medication known to modify seizure threshold, presence of contraindications to transcranial magnetic stimulation (TMS; such as metallic implants in the skull, history of seizures or implanted permanent pacemaker)^20^ or were unable to use an exercise bike. Informed consent was obtained from all participants prior to participation.

Whilst this was a pilot study, a power calculation was performed based on an estimated effect size. To achieve a power of 80%, an allowable difference of 0.5, and population variance of 0.9 at *p* < 0.05, 15 participants were required per group (total sample size of 30 participants). A total sample size of 30 participants has been consistently documented as sufficient for a two-armed pilot study^21^. To be conservative, we aimed to recruit 34 participants to account for possible drop-outs. This study was approved by the University of South Australia’s Human Research Ethics Committee (203,568) and registered with open science framework (https://doi.org/10.17605/OSF.IO/ZE6V7; date of registration 14/7/21) and the Australian New Zealand Clinical Trials Registry (ACTRN12623000339651, date of registration 31/3/23). All methods were performed in accordance with the relevant guidelines and regulations.

### Study design and protocol

**Figure 1:**
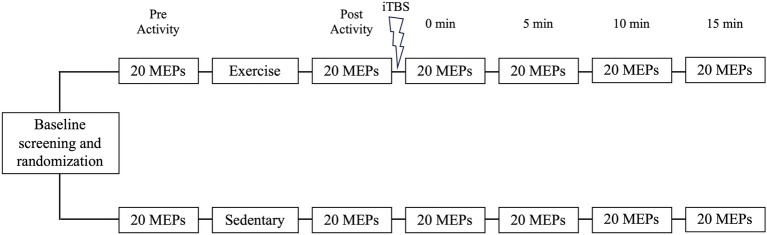
Experimental protocol. Following screening and randomization, procedures are shown with top describing the intervention arm, and bottom the control arm. Abbreviations: iTBS, intermittent theta burst stimulation; MEPs, motor evoked potentials.

A single-blinded, randomized, parallel group, controlled study was conducted to explore physiological effects of cardiovascular exercise on the brain. Participants were allocated to either the intervention or control group following consent using a random number generator. Allocation was concealed prior to enrolment, with intervention personnel and participants unaware of the allocation until all baseline measures were complete. All experimental work was completed in a single session that involved baseline neurophysiological measures of corticospinal excitability, an exercise or rest condition (randomized 1:1), and a neuroplasticity assessment. The measure of neuroplasticity was performed using iTBS and assessments of corticospinal excitability (Fig. 1). Given that a lesion can influence the response to TMS, and therefore, the assessment of neuroplasticity, physiological assessments were performed on the contralesional hemisphere.^22–24^ This approach to physiological testing also ensures a more representative sample of people with stroke. Had we conducted TMS assessments on the ipsilesional hemisphere, our inclusion criteria would have required participants to have recordable responses from the ipsilesional hemisphere, likely limiting our sample to a cohort of well recovered patients^25–27^. The protocol was identical for both groups apart from random allocation to intervention (cardiovascular exercise) or control (rest). Due to the nature of the intervention, participants and personnel involved in data collection were unable to be blind. However, outcome assessors and data analysis personnel remained blind to allocation.

### Baseline assessment

Participant demographics and clinical characteristics including age, sex, time since stroke, levels of physical activity and resting motor threshold (RMT) were obtained. Prior physical activity was assessed using the International Physical Activity Questionnaire—Short Form (IPAQ-SF)^28^. The IPAQ-SF consists of seven items that help retrospectively determine an individual’s total weekly physical activity and time spent in sitting.

### Electromyography (EMG)

Surface EMG was used to record MEPs from the first dorsal interosseous (FDI) muscle of the non-paretic hand, with adhesive disposable EMG electrodes positioned in a belly-tendon montage (22 × 34 mm, FIAB, Florence, Italy). Skin overlying the FDI muscle was cleaned using an alcohol wipe prior to electrode application. A ground strap was placed on the wrist. Participants were seated in a standard chair with the contralesional arm resting on their lap in a pronated position.

### Stimulation

Stimulation was delivered using a Neuro-MS/D rTMS device (Neurosoft Ltd. Ivanova, Russia) that was connected to an oil cooled figure eight coil (wing diameter 70 mm). Single pulses were delivered every five seconds to the contralesional motor cortex region. The coil was held tangentially to the scalp with the handle positioned at a 45-degree posterolateral angle. The optimal position (over the scalp) for evoking MEPs in the resting FDI muscle was located by systematically moving the coil in small increments, then marked with a permanent marker to ensure consistency for subsequent stimulation. An automated algorithm obtained RMT, defined as the lowest stimulus intensity to evoke a MEP of 0.05 mV in the relaxed FDI muscle in at least 5 out of 10 consecutive stimulations. Corticospinal excitability was measured by recording MEPs at 120% RMT and measuring peak-to-peak amplitudes. Blocks of 20 MEPs were completed at each time point (pre-activity, post-activity, 0 min post-iTBS, 5 min post-iTBS, 10 min post-iTBS and 15 min post-iTBS) to ensure reliability of MEP amplitude^29^. Multiple MEP assessments within a time course of 15 min after iTBS were obtained as peak facilitation of MEPs is thought to occur within this timeframe^30^. The FDI was chosen to avoid any potential effect of fatigue on the muscle’s response to stimulation, as it was not directly involved in exercise during the intervention.

### iTBS

iTBS was delivered following the control or intervention. The standard 600 pulses iTBS paradigm was used, consisting of three low intensity, high frequency pulses (50 Hz), applied every 200 ms for two seconds, then repeated every 10 s for a total of 190 seconds^19^. The intensity of stimulation was set at 70% of RMT. Participants were asked to relax and avoid contraction of the upper limb muscles during delivery and following iTBS. Coil position was consistently monitored to maintain correct positioning.

### Cardiovascular exercise

Participants allocated to the intervention (exercise) group completed 20 min of moderate intensity continuous aerobic exercise on a Monark RT2 recumbent exercise bike. Participants were monitored to ensure they stayed within 60–80% of maximum heart rate which was calculated using the formula: 208–(0.7 × age)^31^. A pulse oximeter (ChoiceMMed, Beijing Choice Electronic Technology) monitored oxygen saturation and heart rate at 1 min intervals. The rating of perceived exertion (RPE)^32^ was also used to monitor exertion. Cycling resistance was adjusted as needed to control intensity of the exercise.

### Control condition

Those allocated to the control group were seated in a quiet room and watched a documentary of the same duration (20 min) on a television. The documentary was interesting, but not overstimulating. It was ensured participants did not move around but stayed awake and engaged in a sedentary position.

### MRI acquisition and analysis

Anatomical MRI was available for a subset of participants (n = 26). Images were acquired with a Siemens 3 T MAGNETOM Skyra scanner (Siemens, Erlangen, Germany) with a 64-channel head coil. The scan protocol was: T1-weighted image MPRAGE (voxel 1 mm x 1 mm x 1 mm, repetition time (TR) = 2300 ms, echo time (TE) = 2.98 ms, flip angle = 9°); T2-weighted fluid-attenuated inversion recovery (FLAIR; voxel 1 mm × 0.5 mm × 0.5 mm, TR = 5000 ms, TE = 393 ms). Image processing was carried out using FSL (FMRIB Software Library, Oxford, UK). Non-brain tissue was removed using BET. T1 and T2 images were then registered using FLIRT and lesion masks manually traced by an experienced investigator blind to allocation. Lesion masks were used to obtain lesion volume. A weighted lesion load was also obtained as a measure of injury to the ipsilesional descending motor pathways^33^. Weighted lesion load was selected as it adjusts for the narrowing of the corticospinal tract as it descends from the motor cortex to internal capsule. Lesion masks for each participant were registered to standard Montreal Neurological Institute space using FNIRT. The reference corticospinal tract was derived from the John Hopkins University white-matter tractography atlas. For each slice, the overlap between lesion and corticospinal tract was multiplied by the ratio of maximal corticospinal tract cross-sectional area over cross-sectional area of the corticospinal tract at that slice.

#### Data analysis

All statistical analyses were completed using SPSS (IBM Corp., Released 2020, IBM SPSS Statistics for Windows, Version 27.0, Armonk, NY, USA). Significance level was set at *P* < 0.05. Patient characteristics were compared between groups with independent t-tests for age, time since stroke, RMT, baseline MEP amplitude, lesion volume and weighted lesion load. Sex and IPAQ results were compared with Fisher’s Exact test. Changes in MEP amplitude were assessed using a linear mixed model. Assumptions of normality and homoscedasticity of the residuals for each model were assessed visually using quantile–quantile normal plots and fitted vs residual plots. Several models were evaluated with the model that produced the lowest Akaike Information Criterion (AIC) or prevented overfitting of the data selected. Models were constructed with MEP amplitude (log transformed for normality) as the dependent variable with fixed effect of Group (Exercise, Control) and Time (average MEP amplitude for each timepoint; pre-activity, post-activity, 0 min post-iTBS, 5 min post-iTBS, 10 min post-iTBS, 15 min post-iTBS). Time-point, group and time since stroke interaction was also added since capacity for neuroplasticity changes over time after stroke. Covariates of age, RMT, IPAQ-SF result and time since stroke were added to the analysis as previous studies suggest they may influence neuroplasticity^3, 34–36^. The model was re-run to include the subset of participants with MRI data and to add covariates of lesion volume and weighted lesion load. Finally, given the exploratory nature of this work, we investigated the interaction between Time*Group*Time Since Stroke as we have shown that capacity for neuroplasticity is modified in the first weeks after stroke^3^. First, the correlation (intraclass correlation) between the grand average iTBS response (mean of the post iTBS MEPs divided by the mean of baseline MEPs) and the 10 min post iTBS data (where an effect of exercise was observed) to determine whether responses would be consistent irrespective of the approach to investigate this finding. Second, a hierarchical cluster analysis using Ward’s method was conducted. The purpose was to identify data clusters for the plot between the iTBS response and time since stroke. Finally, time since stroke and iTBS response were compared between clusters (one-way ANOVA for three clusters in exercise group and independent t-test for two clusters in control group).

## Results

### Participant demographics and clinical characteristics

**Figure 2:**
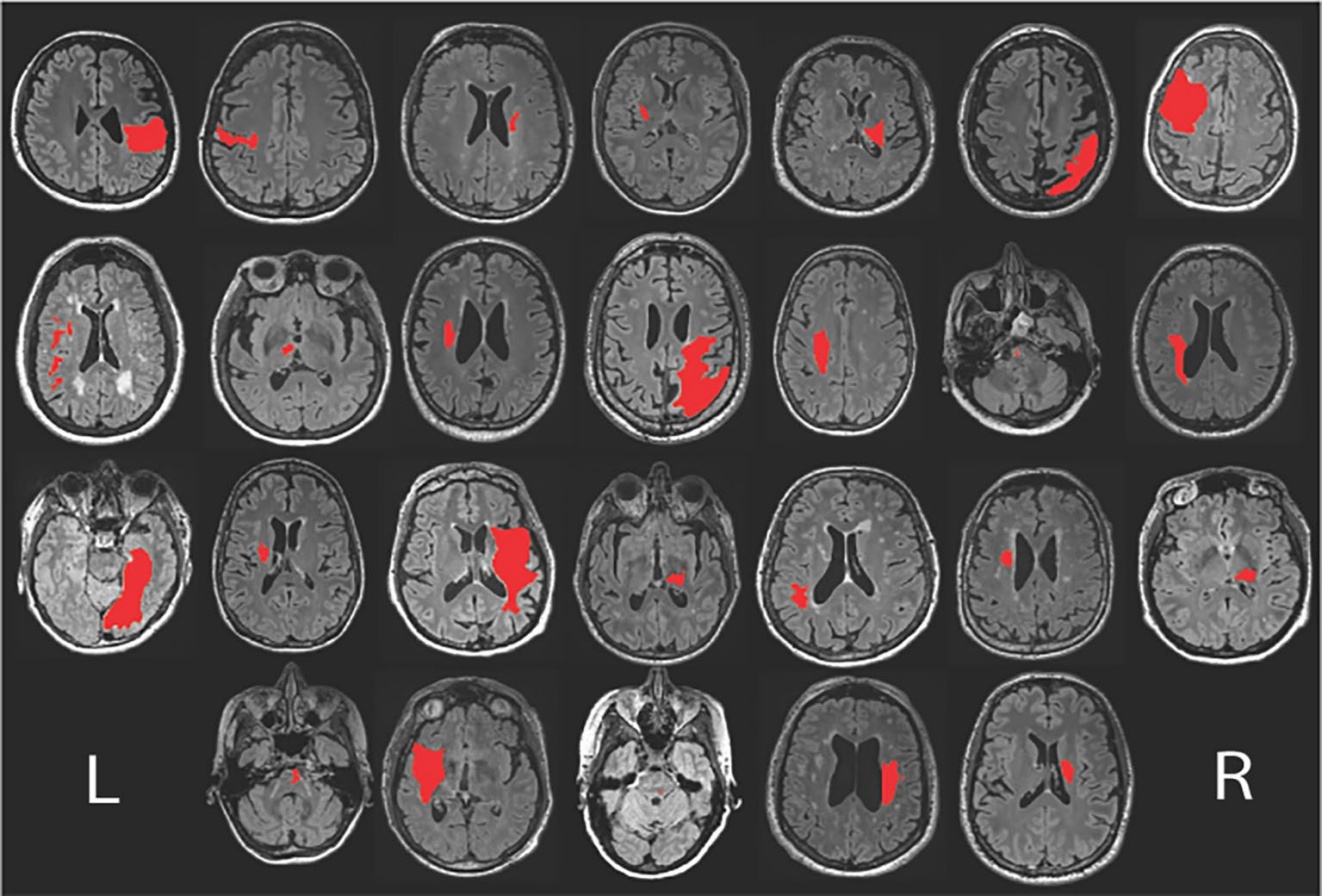
Individual MRI data showing level of greatest cross-sectional area of lesion. Lesion is shown in red.

There were no adverse outcomes reported and all participants completed the study. A total of 33 stroke survivors participated, with 16 randomized to exercise and 17 randomized to the control group. MRI data was available for a subset of 26 participants (11 in exercise and 15 in control). No differences in patient demographics and clinical characteristics were identified between groups (Table 1). Individual MRI data is shown in Fig. 2.

**Table 1:** Participant demographics and clinical characteristics.

	Exercise (n = 16)	Control (n = 17)	Statistics
Age (mean ± SD; years)	62.54 ± 12.64	65.11 ± 7.89	t_(31)_ = 0.71, *P* = 0.48
Sex (n; male)	11	9	*P* = 0.48
Time since stroke (mean ± SD; years)	5.86 ± 4.31	6.38 ± 4.45	t_(31)_ = 0.34, *P* = 0.74
RMT (mean ± SD; %MSO)	42.69 ± 10.46	43.53 ± 10.80	t_(31)_ = 0.23, *P* = 0.82
IPAQ-SF (n; low/moderate/high)	5/10/1	5/11/1	*P* = 1.00
Baseline MEP (mean ± SD; mV)	0.34 ± 0.24	0.41 ± 0.25	t_(31)_ = 0.79, *P* = 0.44
Lesion Volume (mean ± SD; cm^3^)*	17.53 ± 18.56	21.52 ± 32.16	t_(24)_ = 0.37, *P* = 0.72
Weighted lesion load (mean ± SD; cm^3^)*	7.65 ± 9.29	11.88 ± 11.97	t_(24)_ = 0.98, *P* = 0.34

IPAQ-SF, International Physical Activity Questionnaire Short Form; MEP, motor evoked potential; MSO, Maximum Stimulator Output; mV, millivolt; n, number; RMT, resting motor threshold.

*MRI data only available for 11 participants in the exercise group and 15 participants in the control group.

### Effect of exercise on iTBS response

**Figure 3:**
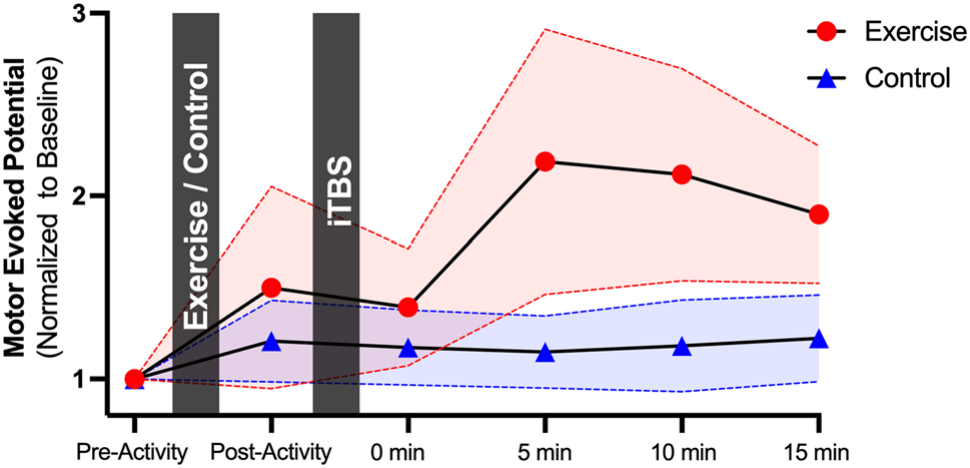
Effect of exercise on iTBS response. X-axis shows time that motor evoked potentials were recorded, and Y-axis provides motor evoked potentials normalized to baseline (values > 1 indicate facilitation of motor evoked potentials by iTBS). Error bars are shown as shaded regions (SEM). The exercise condition appeared to promote a stronger facilitation of motor evoked potentials following iTBS. Abbreviations: iTBS, intermittent theta burst stimulation.

Linear mixed model analyses revealed a significant effect of Group (F_(1, 25)_ = 4.318, *P* = 0.048) and interaction between Time*Group (F_(5, 145)_ = 3.195, *P* = 0.009; see Table 2). Re-analysis of the model to include only the subset of 26 participants with MRI data revealed no effect of lesion volume (*P* = 0.521) or weighted lesion load (*P* = 0.790). Figure 3 provides a graphical summary of the Time*Group interaction, with MEP amplitude normalized to baseline. There appeared to be a greater increase in MEP amplitude following iTBS for the exercise group. This observation was confirmed as MEPs were larger in the exercise group compared to the control group at 10 min post iTBS (t_(31)_ = 1.722, *P* = 0.048), but not at other timepoints (all P > 0.085).

**Table 2:** Results for test of fixed effects.

Source	Numerator df	Denominator df	F	*P* value
Intercept	1	25	3.797	0.063
Time	5	145	0.530	0.753
**Group**	**1**	**25**	**4.318**	**0.048**
**Time x Group**	**5**	**145**	**3.195**	**0.009**
Time x Group x Time since stroke	11	121	1.833	0.055
Time since stroke	1	25	1.607	0.217
Age	1	25	1.673	0.208
Sex	1	25	0.840	0.368
RMT	1	25	0.562	0.461
IPAQ-SF	1	25	0.000	0.995

df, degrees of freedom; RMT, resting motor threshold; IPAQ-SF, International Physical Activity Questionnaire Short Form.

Bold indicates statistical significance.

**Figure 4:**
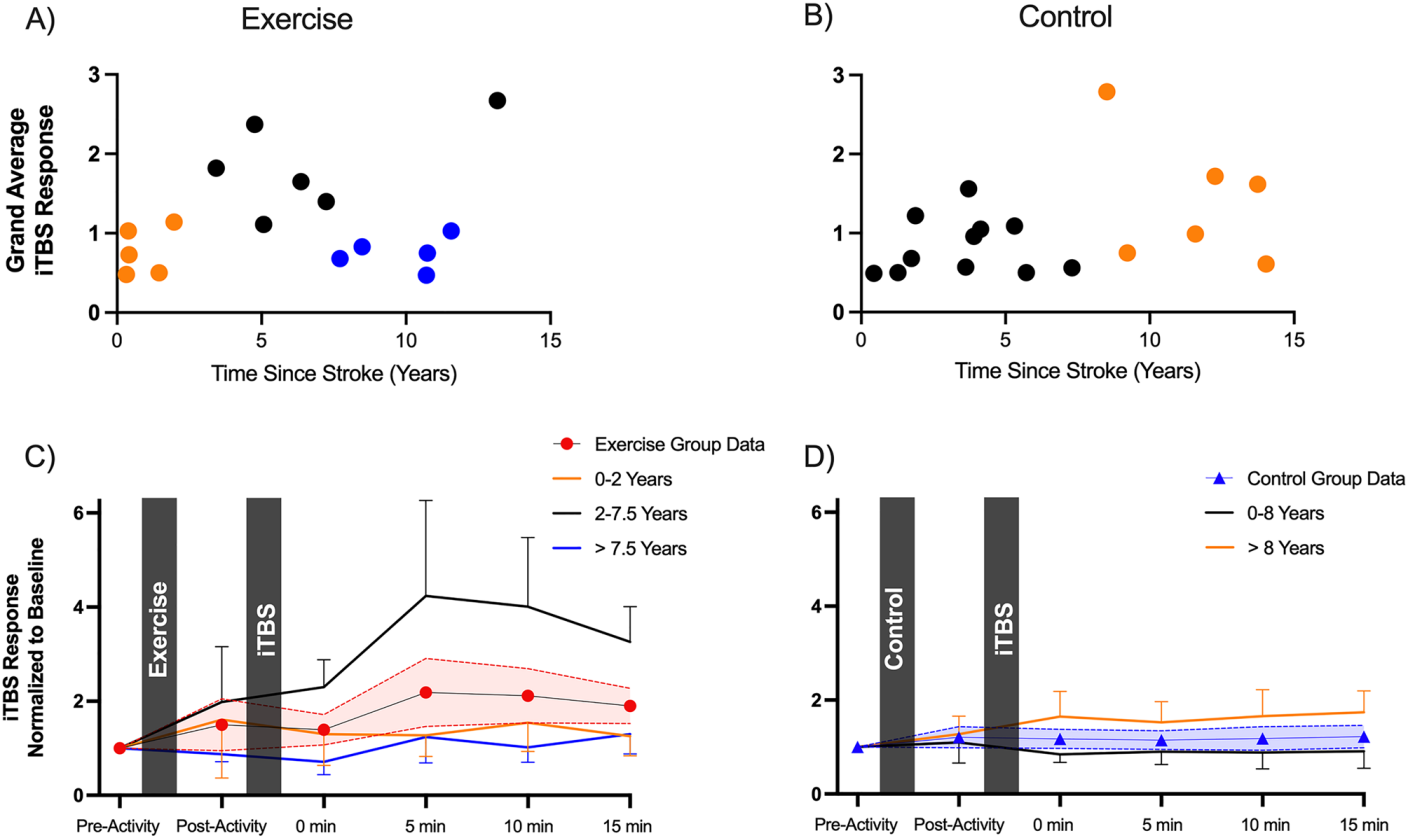
Exploration of the effect of time since stroke on modifying iTBS response following exercise. Top figures show grand average iTBS responses on the Y-axis (values > 1 indicate facilitation of motor evoked potentials by iTBS) and time since stroke on the X-axis for exercise and control groups respectively. For the exercise group (**A**), a hierarchical cluster analysis identified three distinct groupings in the data (shown as orange, black and blue data points). For the control group (**B**), a hierarchical cluster analysis identified two distinct groupings (shown as black and orange data points). Based on cluster analysis, iTBS responses were separated into 0–2 years post-stroke, 2–7.5 years post-stroke and > 7.5 years post-stroke for the exercise group. For the control group, data were separated as 0–8 years post-stroke and > 8 years post-stroke. Bottom figures show effect of exercise (**C**) or control (**D**) on iTBS response. Group averages are shown as per Fig. 3, with data further separated out based on time since stroke in determined by cluster analysis. It appeared that participants who were 2–7.5 years post stroke (black line) exhibited a stronger iTBS response following exercise. Error bars are SEM. Abbreviations: iTBS, intermittent theta burst stimulation.

There was a non-significant trend for the interaction between Time*Group*Time Since Stroke (F_(11, 121)_ = 1.833, *P* = 0.055). Although not reaching statistical significance, we performed a preliminary exploration of this interaction given the pilot nature of this work. First, we plotted the grand average iTBS response (mean post iTBS MEPs divided by mean baseline MEPs) and time since stroke for both the exercise (Fig. 4A) and control groups (Fig. 4B). We selected this approach as a simple way to visualize the iTBS response over time since stroke. Alternatively, plotting the 10 min post iTBS data produced similar findings as this response was highly correlated to the grand average iTBS response (ICC = 0.94, *P* < 0.001). Data were objectively grouped with a hierarchical cluster analysis, identifying three distinct clusters for the exercise group and two clusters for the control group. For the exercise group, these clusters had different time since stroke (F_(2, 13)_ = 18.46,* P* < 0.001) and iTBS responses (F_(2, 13)_ = 12.39, *P* < 0.001). The mean time since stroke of cluster 1 was 0.91 (range 0.32–1.97) years, cluster 2 was 6.67 (range 3.43–13.16) years and cluster 3 was 9.84 (range 7.71–11.56) years. For simplicity of presenting the Time*Group*Time Since Stroke trend, we separated time since stroke as 0–2 years, 2–7.5 years and > 7.5 years (Fig. 4C). Difference in iTBS response appeared to be driven by a stronger MEP facilitation for those participants who were between 2 and 7.5 years post-stroke. For the control data, time since stroke differed between clusters (t_(15)_ = 7.335, *P* < 0.001), but iTBS response did not (*P* = 0.147). The mean time since stroke for cluster 1 was 3.55 (range 0.44–7.30) years, and cluster 2 was 11.56 (range 8.51–14.03) years. For graphical presentation, data from the control group were separated as 0–8 years and > 8 years post stroke (Fig. 4D).

## Discussion

This study aimed to investigate whether moderate intensity aerobic exercise could enhance neuroplasticity in people with stroke. Our measure of neuroplasticity was the change in MEP amplitude after administering iTBS. We observed a stronger iTBS response for people allocated to the exercise group, compared to those in the control group. These preliminary findings might suggest that moderate intensity aerobic exercise could be used to enhance neuroplasticity in people with stroke. Given no adverse events were noted, it appears this is a safe method to modify brain activity. Therefore, moderate intensity exercise might be a clinically feasible method to prime the brain for enhanced stroke recovery in people who are months to years post stroke.

### Exercise can increase neuroplasticity

That iTBS response was greater in the exercise group might indicate increased potential for neuroplasticity after moderate intensity exercise. There is evidence that iTBS can induce an effect that resembles long-term potentiation in the human cortex. Pharmacological studies found that administration of NMDA receptor antagonists blocked after-effects of iTBS, suggesting increases in excitability following iTBS might be due to short-term changes in efficacy of synaptic connections^19, 37^. The effect of iTBS to the motor cortex can be quantified by applying single pulse TMS to trans-synaptically activate pyramidal neurons and record evoked potentials, with amplitude of the MEP providing an indication of corticospinal excitability. Thus, the change in MEP amplitude following iTBS provides an indication of the long-term potentiation like effect on efficiency of synaptic connections in the cortex. The greater the increase in MEP amplitude, the larger the change in synaptic plasticity. While we recorded responses from the contralesional hemisphere, this finding may still be important for promoting neuroplasticity to facilitate stroke recovery. It is reasonable to anticipate that effects of a cycling task are broadly similar between hemispheres^10^, suggesting that responses obtained from the contralesional motor cortex provide a reasonable surrogate of physiological changes in the brain. However, even if this assumption was false, there is evidence that the contralesional hemisphere plays a role in stroke recovery^38, 39^, suggesting upregulation of neuroplasticity in this hemisphere may still contribute to recovery.

Although not evaluated here, BDNF is one possible mechanism that may underpin increased neuroplasticity following exercise. BDNF appears to have a role in regulating synaptic plasticity through both structural and functional effects that act on excitatory and inhibitory synapses in many brain regions^40^. The role of BDNF in supporting synaptic plasticity is evident in preclinical studies where tetanic stimulation to promote long-term potentiation in hippocampal slices was impaired in BDNF knockout mice, but restored by infusion or re-expression of BDNF^41–43^. That an acute bout of moderate intensity exercise can temporarily upregulate hippocampal BDNF expression in rats might suggest a role for exercise in promoting neuroplasticity^44^. Although effects of exercise on BDNF expression are well characterized for the hippocampus, there also exists evidence of BDNF upregulation in the cortex, spinal cord and cerebellum^45^. Similar responses are seen in humans, where serum BDNF increased by 30–40% following a sustained period of 20–40 min of moderate intensity exercise^46^. It may be that the moderate intensity exercise paradigm in this study increased BDNF expression, promoting greater synaptic plasticity as measured with iTBS.

Our findings are well aligned with previous studies. For example, in a small sample of 16 patients with stroke, a 20-min bout of high-intensity interval cycling led to a greater long-term potentiation like effect measured with paired-associative stimulation^47^. However, low intensity cycling did not modify neuroplasticity, as measured with iTBS in a small cohort of 12 chronic stroke survivors^9^. Furthermore, several studies have investigated GABAergic inhibition, a mediator of neuroplasticity, after exercise. In a recent systematic review summarizing this body of work it was reported that a single session of moderate to vigorous physical activity might modify GABA activity^48^. Our findings build upon this past work by quantifying synaptic plasticity in one of the larger studies to be conducted. With the addition of our findings, it appears moderate to high intensity exercise could be beneficial for neuroplasticity in people with stroke, while low intensity exercise may have no benefit. This points towards exercise intensity being important for induction of neuroplasticity. Of course, the behavioral implications of these physiological changes was not tested here, but past studies indicate that moderate intensity exercise promotes maintenance of motor performance during skill acquisition in healthy adults^49^, and improved motor learning and retention in people with stroke^50^. Of importance, there is some evidence to suggest the magnitude of change in neurophysiological measures correlates with improved behavior. In healthy adults, reduced GABAergic inhibition following moderate intensity exercise was correlated with improved motor sequence learning^51^. It might be that the upregulation in neuroplasticity observed in this study could prove beneficial for stroke recovery.

### Time since stroke and effects of exercise on neuroplasticity

A noteworthy finding from this study was the possible role of time post-stroke on neuroplasticity following aerobic exercise. While this outcome should be interpreted cautiously, given it was exploratory, and the sub-group analysis was performed on a small sample, it remains possible that chronicity might influence effects of exercise. Our results appear to suggest a greater response in those who were approximately 2–7.5 years post stroke. While it is unclear why the effect of exercise appeared weaker earlier after stroke (< 2 years), a possible explanation might be that we have interfered with the spontaneous upregulation in neuroplasticity thought to occur after stroke. The precise temporal characteristics of this upregulation in neuroplasticity are not clear, but its occurrence has been physiologically observed in the contralesional hemisphere in human stroke survivors^3^. However, behavioral evidence suggests the critical window for recovery might extend beyond one-year^52^, which could point toward a more persistent, longer-lasting, period of enhanced neuroplasticity. If neuroplasticity was already enhanced, further upregulation may not be beneficial due to effects of metaplasticity. Potentiation/depression induction thresholds are known to adjust in an attempt to maintain stability amongst neuronal networks^53^. If LTP-like processes are heightened and activated repeatedly, the effect may lead to a protective reversal to prevent over-excitation. In support, recent trials or consensus papers on neuroplasticity promoting interventions for stroke recovery suggest later stages of recovery may be better therapeutic targets^53, 54^. While this might suggest that exercise to promote neuroplasticity is better suited to later stages of recovery, we note those who were very chronic (> 7.5 years) demonstrated a weaker response to aerobic exercise. Further investigation is required, but this might reflect long-term neuroanatomical changes, including widespread brain atrophy, shown to continue many years after stroke^55^. In support, there is evidence of an accelerated and persistent decline in cognitive function many years after stroke^56^. Reduced neural substrate could limit both effects of exercise on brain function and our measure of neuroplasticity. Of course, this does not discount other health benefits of cardiovascular exercise and we emphasize caution is needed not to over-interpret this preliminary trend for an effect of time since stroke.

### Alternative explanations

Although the exercise group did exhibit a stronger response to iTBS, it is noteworthy that there was no iTBS facilitation of MEPs in the control group. This was not unexpected. Nonresponse to iTBS is commonly reported in the literature^9, 57, 58^. However, this raises possibilities of alternative explanations for our findings. First, it is possible that exercise attenuates the non-responsiveness of iTBS, rather than increasing the size of iTBS response. The implications of this alternative view would be subtly different with moderate intensity exercise increasing capacity for neuroplasticity to occur, rather than increasing the magnitude of neuroplasticity. Both outcomes might still have potential to benefit stroke recovery. To tease apart these differences, it might be possible to leverage the stronger intra-individual reliability of iTBS response^59^. With relatively robust iTBS responses at repeated sessions, it may be worth having stroke survivors complete both an exercise and control condition. iTBS response it the control condition could then be dichotomized into responder or non-responder to determine if exercise then attenuates non-responsiveness to iTBS, or increases magnitude of response. Second, the increase in MEPs following iTBS in the exercise group could be viewed as a delayed effect of exercise. However, several lines of evidence suggest this is unlikely. First, it is important to keep in mind that the exercise task was cycling, with the upper limbs predominantly remaining at rest. There is evidence that similar paradigms do not lead to increased excitability. For example, a low/moderate intensity cycling task had no effect on MEPs recorded at the paretic hand for up to 30 min after exercise^9^. At higher exercise intensities, there is some indication of increased ipsilesional excitability immediately after exercise^60^, but several studies have reported no effect on contralesional excitability for moderate or high intensity exercise^60, 61^. For these reasons, we suggest it is unlikely our findings reflect a delayed increase in MEPs after exercise.

### Clinical implications

Persistent disability after stroke is an unresolved problem. While much recovery happens in the weeks to months following stroke, likely underpinned by a spontaneous upregulation in neuroplasticity^3^, many people endure ongoing motor impairment. Opportunities to promote neuroplasticity therefore have value in maximizing stroke recovery. Our findings suggest moderate intensity cardiovascular exercise may upregulate neuroplasticity. Given relative safety and simplicity of performing moderate intensity exercise, we suggest this approach is clinically feasible and holds promise as a therapy adjuvant to promote better recovery.

### Limitations and future directions

Findings from this study should be considered with respect to several limitations. First, consistent with the pilot nature of this work, the sample size was relatively small. Future studies should seek to replicate these findings in a larger patient group. Second, there are many factors known to affect response to brain stimulation protocols^54, 62–65^. While most were controlled through the inclusion criteria or statistical analysis, it remains possible that diurnal variations in cortisol^64^, genetic profiles^64^, or intrinsic properties of the stimulated network^62, 65^ might have had some influence on our results. Third, the effect of exercise on iTBS was most strongly observed at 10 min which was expected as peak MEP facilitation is known to occur within this timeframe^30^. While this might suggest exercise can increase neuroplasticity, we do not have the temporal resolution in our data to know whether exercise could prolong effects on neuroplasticity. It appears peak facilitation of MEPs was beginning to dissipate after 10 min in the exercise group, but there could be benefits in future studies exploring how long benefits of exercise on neuroplasticity might persist. Fourth, this study did not include an assessment of patient impairment or activity level. This may have been beneficial to: (1) further interpret IPAQ physical activity levels relative to motor impairments, (2) identify the level of activity or impairment required to participate in moderate intensity exercise, enabling replication of the intervention, and (3) explore individual characteristics that might modify the physiological effect of exercise. Future studies should consider inclusion of impairment and activity measures. Finally, this study only evaluated the effects of an acute, 20-min, moderate intensity exercise session on a physiological assessment of neuroplasticity. We cannot directly infer effects of different exercise intensities, durations, or possible behavioral benefits that could be achieved with subsequent training. Further studies are required to explore optimal parameters of exercise and associated behavioral gains that could be achieved when paired with therapy.

### Conclusion

Our results provide an indication that moderate intensity aerobic exercise might promote neuroplasticity in people with stroke. These promising findings point towards the use of cardiovascular exercise to prime the brain for subsequent training. Therapies that can enhance neuroplasticity might provide opportunity to maximize stroke recovery. Moderate intensity cardiovascular exercise appears worthy of investigation in clinical trials.

## Data Availability

The datasets generated during used and/or analysed during the current study available from the corresponding author on reasonable request.
